# A quantitative method to measure biofilm removal efficiency from complex biomaterial surfaces using SEM and image analysis

**DOI:** 10.1038/srep32694

**Published:** 2016-09-07

**Authors:** N. Vyas, R. L. Sammons, O. Addison, H. Dehghani, A. D. Walmsley

**Affiliations:** 1 Physical Sciences of Imaging for Biomedical Sciences (PSIBS) Doctoral Training Centre, College of Engineering & Physical Sciences, University of Birmingham, Birmingham, B15 2TT, UK; 2 School of Dentistry, Institute of Clinical Sciences, College of Medical and Dental Sciences, University of Birmingham, Mill Pool Way, Birmingham, B5 7EG, UK; 3 School of Computer Science, University of Birmingham, Edgbaston, Birmingham, B15 2TT, UK

## Abstract

Biofilm accumulation on biomaterial surfaces is a major health concern and significant research efforts are directed towards producing biofilm resistant surfaces and developing biofilm removal techniques. To accurately evaluate biofilm growth and disruption on surfaces, accurate methods which give quantitative information on biofilm area are needed, as current methods are indirect and inaccurate. We demonstrate the use of machine learning algorithms to segment biofilm from scanning electron microscopy images. A case study showing disruption of biofilm from rough dental implant surfaces using cavitation bubbles from an ultrasonic scaler is used to validate the imaging and analysis protocol developed. *Streptococcus mutans* biofilm was disrupted from sandblasted, acid etched (SLA) Ti discs and polished Ti discs. Significant biofilm removal occurred due to cavitation from ultrasonic scaling (p < 0.001). The mean sensitivity and specificity values for segmentation of the SLA surface images were 0.80 ± 0.18 and 0.62 ± 0.20 respectively and 0.74 ± 0.13 and 0.86 ± 0.09 respectively for polished surfaces. Cavitation has potential to be used as a novel way to clean dental implants. This imaging and analysis method will be of value to other researchers and manufacturers wishing to study biofilm growth and removal.

Biofilm formation on biomaterial surfaces may significantly impact on the behaviour or survival of the biomaterial or device. Biofilms are complex microbial communities characterised by cells attached to the substrate surface, to interfaces or to each other and are embedded in an extracellular polymeric matrix which they have produced^1^,^2^,^3^. The behaviour of microbes in a biofilm can differ significantly from the behaviour of the same organism studied in planktonic conditions with respect to growth rate and gene transcription^3^. Clinically biofilms form on native tissues such as skin, oral mucosa and teeth and often cause chronic infection of biomedical devices such as urinary catheters, intravenous cannulas and dental implants^4^,^5^,^6^,^7^,^8^,^9^. Management of biofilm related infections can be problematic as the structure and composition of the biofilm itself offers protection against antimicrobial agents and frequently conjunctive mechanical biofilm disruption is required to enable surface disinfection^10^.

As a consequence, considerable research effort is currently being spent developing methods and instrumentation to remove biofilms from material surfaces. A current deficiency in the research effort is the ability to quickly and objectively evaluate the efficiency of biofilm disruption with a high level of accuracy. Many different methods have been used to assess biofilm removal efficiency. Biological approaches include semi-quantitative staining, measurements of dried biomass, protein or DNA quantification, or assessments of residual viable organisms through standard microbial culture techniques^6^,^11^,^12^,^13^,^14^. Each method has advantages and deficiencies but they all provide only indirect values of the removal efficiency and are prone to operator induced variability.

In contrast, direct imaging of the biofilm provides information on its structural characteristics, its interaction with the surface, as well as spatial information regarding the homogeneity of biofilm disruption. Confocal laser microscopy, electron microscopy, light microscopy, bioluminescence imaging and macroscale photography have all been used as measurements to assess biofilm disruption^5^,^7^,^8^,^9^,^15^,^16^,^17^,^18^,^19^,^20^,^21^,^22^,^23^,^24^,^25^. Frequently however, such techniques are only used qualitatively or semi-quantitatively leading to a high risk of bias in the investigation. When quantitative measurements are made, information such as biofilm area/volume and thickness can be acquired to establish the effectiveness of the intervention^20^,^25^. Segmentation, the method of partitioning an image into segments based on various image characteristics, has been used to calculate the area of the surface covered by biofilm. One common approach is simple thresholding, which is effective when there are differences in the pixel intensity value between biofilm and background. Many studies investigating biofilm removal have used thresholding to segment images of biofilm from photographs and light microscopy images, however typically manual thresholding has been employed^8^,^17^,^26^, which is subjective leading to large errors and making intra-operator reproducibility impossible. Other studies using automatic thresholding have failed to specify the exact method of segmentation or validate its accuracy^7^,^16^,^21^. Moreover, the imaging techniques used in these studies for calculating such data (light microscopy, confocal microscopy and photography) do not give sufficient resolution to image individual bacteria in biofilms. Therefore a gap exists to develop a methodology that can consistently, accurately and objectively segment biofilm from images to quantify biofilm disruption.

Scanning electron microscopy (SEM) has been used extensively for qualitative observation of biofilm disruption due to its high resolution and is usually applied in combination with biological assays of biofilm removal efficiency^5^,^7^,^8^,^18^,^19^,^24^. Using SEM images, simple thresholding often cannot be implemented as the intensity values of the biofilm and normal surface are similar due to the same effective contrast seen by SEM. Rough (textured) biomaterial surfaces such as those found on components intended for osseo-integration further complicate the image analysis, so advanced segmentation methods such as semi-supervised machine learning techniques are typically required^27^.

The overall objective of the study is to develop a work-flow for SEM imaging and quantitative image analysis of biofilm samples before and after mechanical disruption that can be applied to complex surface topologies for a wide variety of biomaterial applications. Methodological development is undertaken using a ‘case-study’ application of biofilm disruption on Titanium (Ti) dental implants which possess a variety of surface topologies ranging from highly polished through to complex tortuous surfaces engineered to promote osseo-integration^28^. Ti dental implants are susceptible to peri-implant inflammation (peri-implantitis) driven by the presence of a surface biofilm and which leads to loss of bony integration and ultimately implant failure^29^,^30^. Removal of biofilm from dental implants is difficult and many methods damage the surface. Cavitation occurs around dental ultrasonic scaler tips which are commonly used for mechanical debridement^31^,^32^,^33^ and cavitation bubbles generate shock waves, acoustic microstreaming and high velocity micro-jets^34^,^35^,^36^ which may be able to disrupt biofilm without modifying the implant surface. The specific aims of this study were therefore to develop novel quantitative methods to assess the effectiveness of mechanical biofilm disruption on biomedical Ti surfaces possessing clinically relevant surface topologies.

In this work we present an imaging and image analysis protocol for calculating the area of biofilm on Ti surfaces from SEM images, before and after application of cavitation from an ultrasonic scaler. We use this to calculate the area of biofilm removed after treatment. We further demonstrate that cavitation is able to disrupt biofilm from sand blasted, acid etched (SLA) and polished dental implant surfaces, showing potential for this technique as a new method of dental implant debridement. The protocol developed to determine biofilm area from SEM images will allow researchers who study biofilm growth and disruption to use SEM as a quantitative tool and will facilitate future breakthroughs in the field of medical implant infection.

## Results

**Figure 1:**
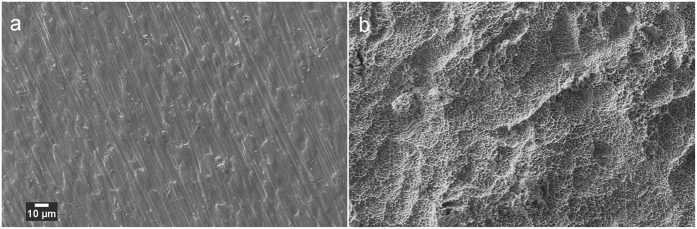
SEM images of the clean surfaces produced in the study, imaged before biofilm colonisation. (**a**) Polished surface (**b**) SLA surface.

**Figure 2:**
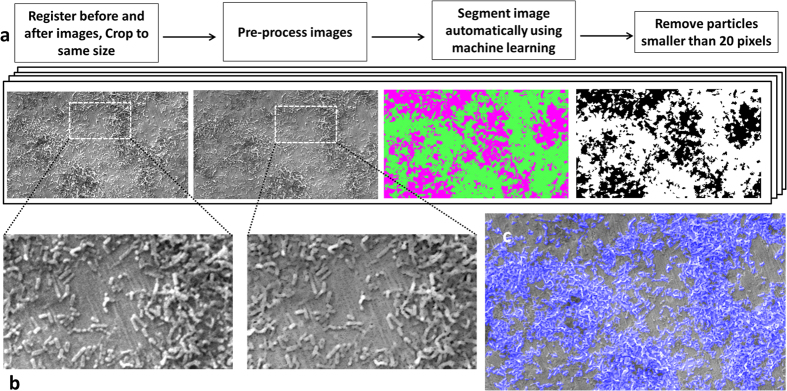
(**a**) Method developed to segment biofilm from the surface in SEM images. (**b**) Close-up showing image before and after pre-processing – outliers were removed to smooth scratches on the surface, for better segmentation. (**c**) Overlay of the segmented area (blue) on the SEM image, showing accurate detection of biofilm.

**Figure 3:**
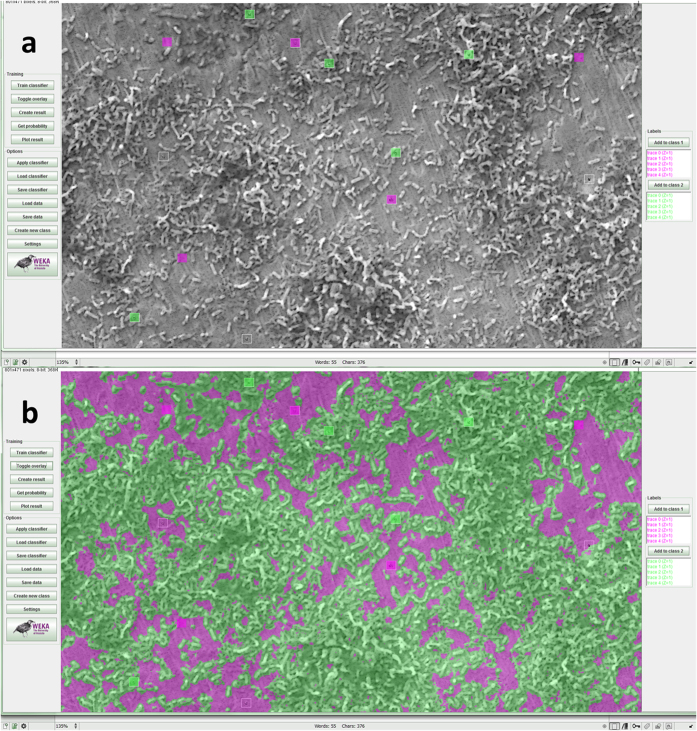
Screenshots of the Trainable Weka Segmentation plugin in Fiji^37^. (**a**) Square regions of interest were classified by the user as samples to train the program to segment the rest of the image, where biofilm is labelled green and the surface is labelled magenta. (**b**) Whole image segmented automatically from the samples given in (**a**). The Weka logo http://www.cs.waikato.ac.nz/ml/images/Weka (software) logo.png> is available under the Creative Commons Attribution-ShareAlike 2.5 http://creativecommons.org/licenses/by-sa/2.5/ License (http://creativecommons.org/licenses/by-sa/2.5/) for the use of the logo in this figure. Please see the terms of use on the Weka website for more information: http://www.cs.waikato.ac.nz/ml/weka/citing.html.

SEM images of the two surfaces prepared on the Ti test specimens are shown in Fig. 1. Culture of *S. mutans* on Ti surfaces under biofilm conditions resulted in typically heterogeneous distributions of biofilm with greater growth observed on roughened ‘SLA’ surfaces. Figure 2 demonstrates the developed methodology to segment biofilm from the Ti surface in SEM images using an automatic machine learning process. Figure 3 demonstrates the use of the Trainable Weka Segmentation plugin whereby baseline user classification of the surface and of the biofilm trains the programme to subsequently segment the image^37^.

**Figure 4:**
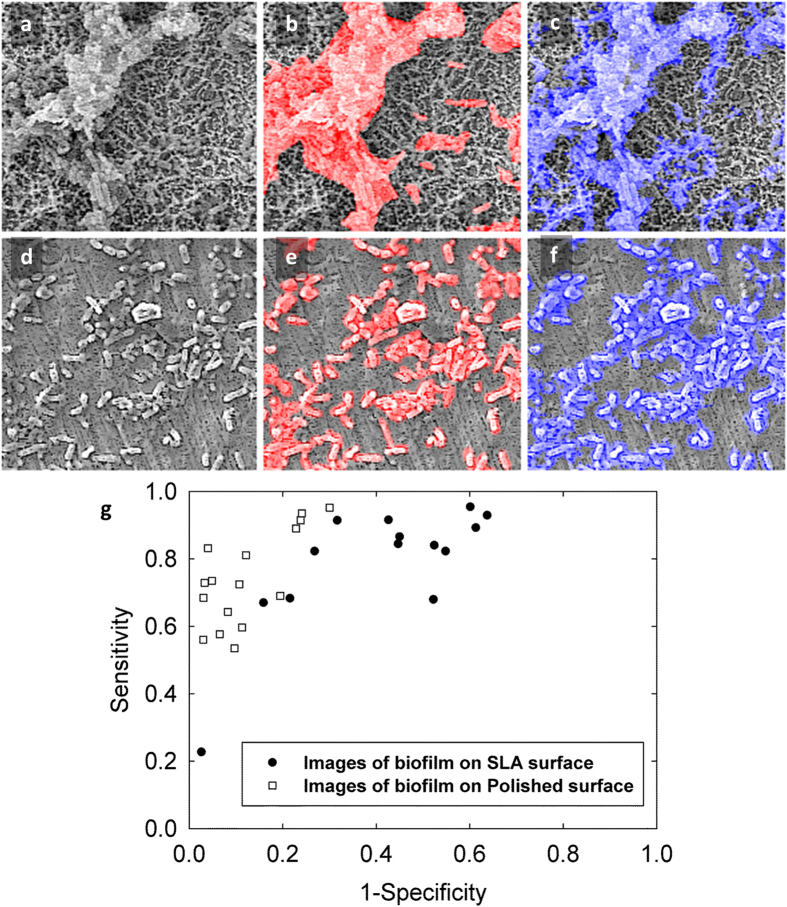
Examples of comparing the automatic segmentation to a manual reference segmentation. (**a**) SLA surface with biofilm growth. (**b**) Overlay in red showing the manual segmentation. (**c**) Overlay in blue showing the automatic segmentation. (**d**) Polished surface with biofilm growth. (**e**) Overlay with manual and (**f**) automatic segmentation. (**g**) Receiver Operator Characteristics curve showing accuracy of segmentation for different images.

**Figure 5:**
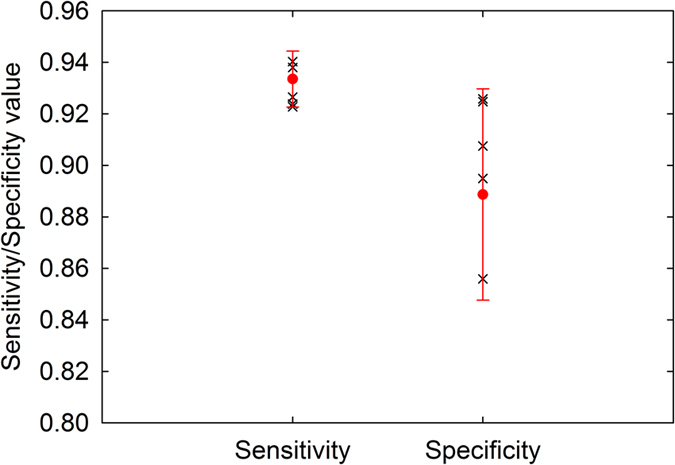
Sensitivity and specificity values obtained when testing the reproducibility of the initial sample pixel choice. All values are over 0.8, showing the method of choosing sample pixels is reproducible.

The sensitivity and specificity of automatic segmentation were calculated by comparison with manual segmentation (manually delineating all bacteria in the image) for both types of surface topology. The mean sensitivity and specificity for segmentation of the polished surface images were 0.74 ± 0.13 and 0.88 ± 0.09 respectively (Fig. 4). The mean sensitivity and specificity values for segmentation of the SLA surface images were 0.80 ± 0.18 and 0.62 ± 0.20 respectively. The receiver operating characteristics curve in Fig. 4 demonstrates the accuracy of segmentation of different images and outlying data was noted only when very few bacteria were present in the image field. ROIs were randomly placed on either the surface or the biofilm in the SEM images by the operator when training the algorithm. The reproducibility of this selection method was tested to determine whether the placement of the ROIs had an effect on the outcome of the segmentation. The mean sensitivity and mean specificity were calculated as 0.93 ± 0.01 and 0.89 ± 0.04, respectively (Fig. 5).

**Figure 6:**
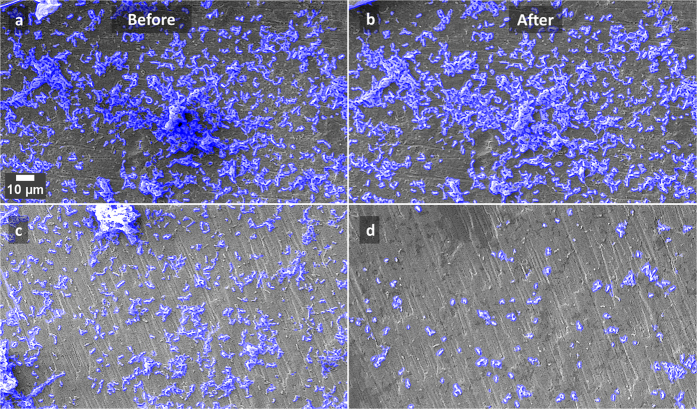
Examples of SEM images of the polished discs, before and after treatment with the ultrasonic scaler held 1 mm away for 30 s, at low power (**a**,**b**) and medium power (**c**,**d**). The blue overlay shows the automatic detection of bacteria.

**Figure 7:**
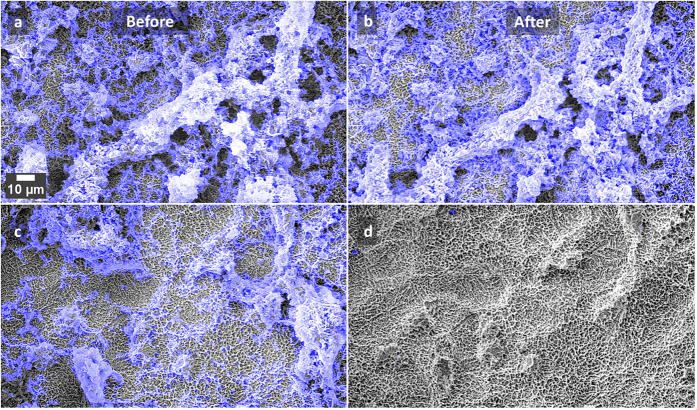
Examples of SEM images of the SLA discs, before and after treatment with the ultrasonic scaler held 1 mm away for 30 s, at low power (**a**,**b**) and medium power (**c**,**d**). The blue overlay shows the automatic detection of bacteria.

**Figure 8:**
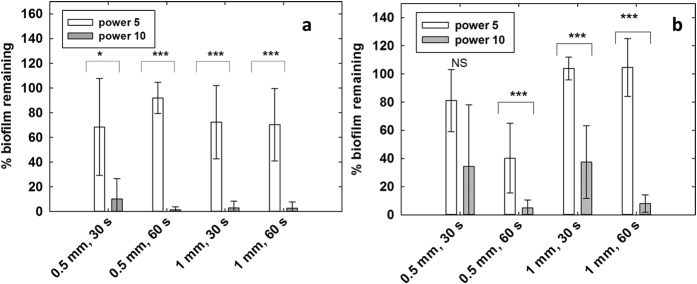
Mean percentage of biofilm remaining calculated from the segmented before and after images, (**a**) SLA surface, n = 10 for each setting. (**b**) Polished surface. n = 8 for each setting. *p < 0.05, ***p < 0.001 (Rank Sum Test).

Application of the developed methodology to the applied ‘case-study’ demonstrated good concordance of the statistical analysis of the quantitative data with assumed behaviour related to the biofilm disruption method, namely greater disruption would be expected with greater cavitation generation (higher power, longer duration of application). There was a statistically significant difference between the amounts of biofilm removed at power 5 and power 10 for all except 1 condition. More biofilm was removed when the scaler was used at medium power compared to low power (Figs 6 and 7). There are also statistically significant differences in the amount of biofilm removed when the scaler was operated for 30 s and 60 s for the following settings: distance 0.5 mm, power 10, SLA discs (p = 0.021), and distance 1 mm, power 10, polished discs (p < 0.001). In these cases more biofilm was removed after operating the scaler for 60 s (Fig. 8).

## Discussion

A large number of studies have reported the impact of experimental variables on biofilm disruption on native tissues and/or biomaterial surfaces but have used qualitative methods or methods that have high susceptibility to measurement bias. As a consequence the interpretation of findings can be limited impacting on technique development or adoption. The current study reports the development and validation of an image analysis protocol which can be used to quantify biofilm disruption on different surface topologies.

In contrast to previous studies which have used basic thresholding techniques to segment biofilm from low magnification microscopy images, the segmentation technique employed in this study uses machine learning, where a wide range of feature detection techniques including texture filters and edge detectors are used to train the algorithm from the image. As the method does not rely only on intensity differences in the image, it can be applied to segment SEM images where the background is non-uniform, such as tortuous biomaterial surfaces.

Another difference of this method is that no proprietary software is used; all image segmentation is done using the open source Fiji image processing package.

We have also tested the accuracy and the reproducibility of the method, which previous studies have not done. The ROC curve shows that segmentation was more accurate on the images of the polished discs, as expected, due to easier detection both automatically and manually as there was more contrast between the biofilm and background, and the surface was less irregular.

The accuracy of the segmentation of the SLA surface was lower (mean specificity 0.62 ± 0.20 and mean sensitivity 0.80 ± 0.18). This implies that the algorithm often detected bacteria where the reference segmentation labelled the area as surface. However, it should be noted that although manual segmentation is the gold standard and was therefore used as the reference segmentation, there were cases for the SLA samples where even an experienced operator may have inevitably incorrectly identified a few individual bacteria as surface. This does not invalidate the method. This occurred due to pixellation in the image making it difficult to resolve the individual bacteria (e.g. bottom right of Fig. 4b,c). There is an outlier on the ROC curve where the specificity is high (0.9) but the sensitivity is low (0.2). In this case it was difficult to identify a raised section in the image as biofilm/surface, and the operator labelled it as biofilm whereas the algorithm detected it as surface. Therefore it could be useful in similar cases to also take images in back-scattered electron mode and to perform energy dispersive X-ray spectroscopy to determine the chemical composition. The magnification of x1250 used in this study is sufficient to image and segment biofilm however if individual bacteria need to be segmented then a either a higher magnification or a higher resolution would be required. Overall, the algorithm struggled to segment correctly in the same situations where the human operator struggled to segment correctly, and the lower sensitivity and specificity values are due to this.

Further work can be done to improve the accuracy of the method, for example by using other types of classifiers from the Weka Package manager and comparing the performance of different types of classifiers.

The graphs in Fig. 8 suggest that more biofilm removal occurred from the SLA surfaces compared to the polished surfaces, which is an interesting finding. This result could be because the cavitation bubbles are more effective on the roughened surface due to its microgrooves, which allow more cavitation nuclei to form. Further work (e.g. using high speed imaging) can show the cavitation dynamics in more detail. Research on different oral tissue and biomaterial surfaces would also show the effect of cavitation on biofilm removal more effectively. The protocol we have developed may be used to determine this.

The protocol for measuring the area of biofilm removal through the use of SEM finder grids and image processing is a novel application for quantitative analysis of SEM images before and after an experimental treatment.

We have used this method to show that cavitation from ultrasonic scalers is effective at cleaning biofilm from dental implant surfaces. This could lead to ultrasonic scalers being used in a novel non-contact mode for treating peri-implant mucositis and peri-implantitis.

This method also has potential to be used to evaluate biofilm growth on a surface over time, and it is not only limited to SEM images, but could be used to segment biofilm from other types of microscopic images.

To the best of our knowledge, we are the first to propose a method for accurately segmenting biofilm from SEM images to calculate the percentage of biofilm removed/remaining. This method can easily be applied to other studies to enable researchers to use SEM as both a qualitative and quantitative tool for evaluation of biofilm disruption.

## Methods

### Preparation of the biomedical Ti substrate

The following protocol from Yu *et al*. was used to replicate dental ‘SLA’ (Sandblasted, Large-grit, Acid-etched) implant surfaces^38^: 16 commercially pure ASTM Grade II Ti discs of 14 mm diameter and 1 mm thickness (Titanium Products Ltd, Birmingham, UK) were polished manually (DAP-7, Struers, Ballerup, Denmark) using P500 SiC paper and then sandblasted with 250 μm corundum particles (Korox, BEGO) (SANDIMAT, Local exhaust ventilation, Allianz Engineering Inspection Service Ltd, Italy). Discs were then cleaned in distilled water (dH_2_O) for 15 mins in an ultrasonic bath, and etched in 37% HCl/98% H_2_SO_4_ (1:1) at 80 °C for 5 mins. A further 16 Ti discs were polished to a high surface finish to mimic the surface generated at the superficial ‘collar’ region of dental implants using sequentially increasing grades of SiC abrasive papers from P500-P2000 for standardised time periods using dH_2_O as the lubricant. The prepared discs were then sequentially cleaned in acetone, ethanol and dH_2_O in an ultrasonic bath for 15 mins each and subsequently sterilised by autoclaving at 121 °C for 1 h.

### Bacterial cell culture and biofilm generation

The Gram-positive bacteria *Streptococcus mutans* (strain 3209) was used in the current study to form a simplistic early biofilm model for the development of the image analysis techniques. Briefly, the stock microorganisms were recovered from porous storage beads maintained at −20 °C and initially grown on the Tryptone Soya Agar (Oxoid, UK) media for 24 h. The *S. mutans* culture was transferred to a sealed CDC biofilm reactor Model CBR 90-3-DH-int (Biosurface Technologies Corp, Montana, USA) into which the Ti discs were suspended vertically in a bespoke holder with Tryptone Soya Broth (Oxoid, UK containing 25% Sucrose (Sigma Aldrich, USA) as the media. After 24 h in batch mode the fed batch (continuous flow) of incubation was then commenced by turning on a peristaltic pump for 96 h. Agitation of the culture media was maintained throughout using a magnetic stirrer at 100 rpm. The Ti discs were removed from the biofilm reactor after a total of 5 days of incubation and then fixed in 0.1 M sodium cacodylate buffer and 2.5% glutaraldehyde (25% EM grade, Agar Scientific, Essex, UK), dehydrated using serial ethanol gradient immersions and then gold sputter-coated (Emitech K550X, Kent, UK) for SEM as previously described^39^. Copper SEM finder grids (Agar Scientific, UK) of 10 mm diameter and a mesh size 450 × 450 μm were attached to the discs to aid in locating the same areas before and after biofilm removal (Figure S1).

### Biofilm removal

Biofilm was removed with a Satelec P5 Newtron ultrasonic generator with tip 10P (Satelec, France). The tip was immersed in distilled water (dH_2_O) with the water flow from the scaler turned off (Figure S1). The following setting combinations were used: power 5 or 10 (corresponding to low and medium power); held 0.5 mm or 1 mm away from disc; scaler operated for 30 s or 60 s. The removal process was also imaged with a high speed camera (HPV-1, Shimadzu Corporation, Japan) at 128,000 fps to visualise cavitation (Supplementary Figure S2).

### SEM imaging

A Zeiss EVO MA-10 scanning electron microscope was used for imaging. Ti discs were placed into the SEM sample holder at the same orientation before and after removal so the same area could be identified and standardised. Imaging was performed at x100, x500 and x1250 magnifications at a working distance of 5–8 mm to aid in locating the same area after biofilm removal. Images taken at x1250 magnification were used for analysis (Figure S1). Biofilm was imaged in areas where the Ti surface was also visible before removal, so features in the surface could be used as markers for accurate co-registration.

### Image analysis

All image analysis was performed using Fiji (distribution of the ImageJ software, US National Institutes of Health, Bethesda, Maryland, USA) according to the following methods^40^. The pixel size of SEM images used in the analysis was 250 nm.

### Image registration

SEM images of biofilm taken in similar locations on the Ti discs before and after removal were firstly co-registered (aligned) so the same area was observed in both images. Registration used the ‘Register virtual stack slices’ plugin^41^, with a rigid feature extraction model and a rigid (translate and rotate) registration model. The shrinkage constrain option was employed so a reference image did not need to be chosen. The maximal alignment error of the geometric consensus filter was set to 8 pixels for more precise registration, and the default settings were used for the feature descriptor and scale invariant interest point detector. Images were then cropped to the same size to enable batch processing in the segmentation step and ensured that blank pixels (due to the image being translated and rotated during the registration procedure) were removed.

### Pre-processing for SLA surface images

Different pre-processing steps were conducted according to the surface topology (rough SLA surface and the polished surface) to enable more accurate segmentation. For images of biofilm on the SLA surface, a rolling ball background subtraction was applied (1000 rolling ball radius, sliding paraboloid) to correct for uneven illumination which occurred due to the rough texture of the surface which had microscale pits due to the exposed grain boundaries resulting from acid etching. The contrast was enhanced by 1% and the Remove Outliers filter was applied using a threshold of 50 and radius of 2 to remove any bright pixels on the SLA surface for easier segmentation. This filter replaced any pixels greater than 50 with the median value of the surrounding area (circle of radius 2). The gamma operation was applied with value 0.6 for non-linear histogram adjustment before sharpening the images to obtain improved contrast between individual bacteria and the surface.

### Pre-processing for polished surface images

For images of biofilm on the polished surface, contrast was enhanced by 0.4% and the Remove Outliers filter was applied with a threshold of 0 and a radius of 2, so that all pixels were replaced by the median of the surrounding area (circle of radius 2). This ‘smoothed’ the scratches in the surface so they would not be misinterpreted as biofilm in the segmentation step.

### Segmentation

The biofilm was segmented from the surface using the Trainable Weka Segmentation plugin^37^ which utilises a collection of machine learning algorithms for segmentation. Specifically, this algorithm computes features from the input image using edge detectors, texture filters, noise reduction filters and membrane filters to create feature vectors, which are then applied to the learning algorithm. Pixel samples are provided by the user, and a classifier is trained using these samples in a semi-supervised way. The trained classifier can then be applied to segment the rest of the image(s) automatically. The default classifier (Fast Random Forest) was used for all analysis in this study.

The pixel samples chosen by the user for training can be free-drawn, for example with the lasso tool in Fiji, but to ensure consistency and reproducibility a set of square regions of interest measuring 12 × 12 pixels were used. This size was optimal as it was large enough to provide enough pixel values for training and small enough to fit over small clusters of biofilm and individual bacteria. Around 10 to 20 regions of interest (ROIs) were placed on the image by the user and put into one of two classes - biofilm or surface. If needed, the ROIs were repositioned by the user to retrain the classifier for improved segmentation. The following training features were chosen to create a classifier for the majority of images: Gaussian blur, Sobel filter, Hessian, Difference of Gaussians, Membrane projections, Variance, Mean, Minimum, Maximum, Median and Bilateral, as this combination gave the most effective segmentation in the least amount of time (approximate computation time: 10 s per image). All of the training features available were chosen for segmenting the SLA images with less bacteria as they were more difficult to segment (approximate computation time: 120 s per image) (in addition to the features mentioned above, the following were also used: Anisotropic diffusion, bilateral, Lipschitz, Kuwahara, Gabor, Derivatives, Laplacian, Structure, Entropy, Neighbours). Other settings were kept as default (membrane thickness 1, membrane patch size 19, minimum sigma 1.0, and maximum sigma 16.0).

For certain images, batch processing was possible so a classifier only needed to be trained once and could then be applied to the rest of the images to automatically segment them. This could be done for all of the images of the polished discs - in this case two classifiers were trained, one for images with more than half of the surface covered by bacteria and secondly for images with less than half the surface covered by bacteria.

Batch processing was possible for some images of biofilm on the SLA surface - two classifiers were again trained as before, and any images which could not be segmented by these classifiers were segmented separately, with a separate classifier being trained for each image. In some cases, images needed further brightness and contrast adjustments for accurate segmentation.

### Post-processing

Objects smaller than 20 pixels were assumed to be noise and were removed from the segmented images using the ‘Analyse Particles’ plugin. The binary images were inverted if necessary so the surface was black and the biofilm areas were white. The histogram of each image was calculated to find the number of white pixels, corresponding to the biofilm area. The percentage of biofilm remaining on the surface after instrumentation was calculated:


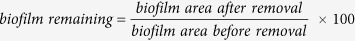


### Statistical analysis

The Mann-Whitney Rank Sum Test was used for testing for statistical significance, with significance defined as α = 0.05. SigmaPlot 12.3 (Systat Software, USA) and MATLAB (The Mathworks Inc, USA) were used for analysis and data plotting. The accuracy of the segmentations was evaluated by comparing to a manual segmentation done by the user, for one pair of images for each setting measured. (16 pairs of images in total), and calculating the sensitivity and specificity using a custom written MATLAB script. The regions of interest chosen by the operator for training the classifier were randomly selected. To test the reproducibility of this method, a classifier was trained 6 times on one image where 10–20 sample ROIs were placed in different locations each time, resulting in 6 segmented images. These were compared to a reference image segmented with a classifier trained with 40 sample ROIs, and sensitivity and specificity were calculated.

## Additional Information

**How to cite this article**: Vyas, N. *et al*. A quantitative method to measure biofilm removal efficiency from complex biomaterial surfaces using SEM and image analysis. *Sci. Rep*. **6**, 32694; doi: 10.1038/srep32694 (2016).

## Supplementary Material

Supplementary Information

Supplementary Video S1
