# Supplementary material for: A quantitative method to measure biofilm removal efficiency from complex biomaterial surfaces using SEM and image analysis
Source: Sci Rep. 2016 Sep 7;6:32694. doi: 10.1038/srep32694 (PMC5013386; doi:10.1038/srep32694)
Supplement: Supplementary Information [file srep32694-s1.pdf]

A quantitative method to measure biofilm removal efficiency from complex biomaterial surfaces using SEM and image analysis

N. Vyas[1,2], R. L. Sammons[2], O. Addison[2], H. Dehghani[3], A. D. Walmsley[2*]

Supplementary Information

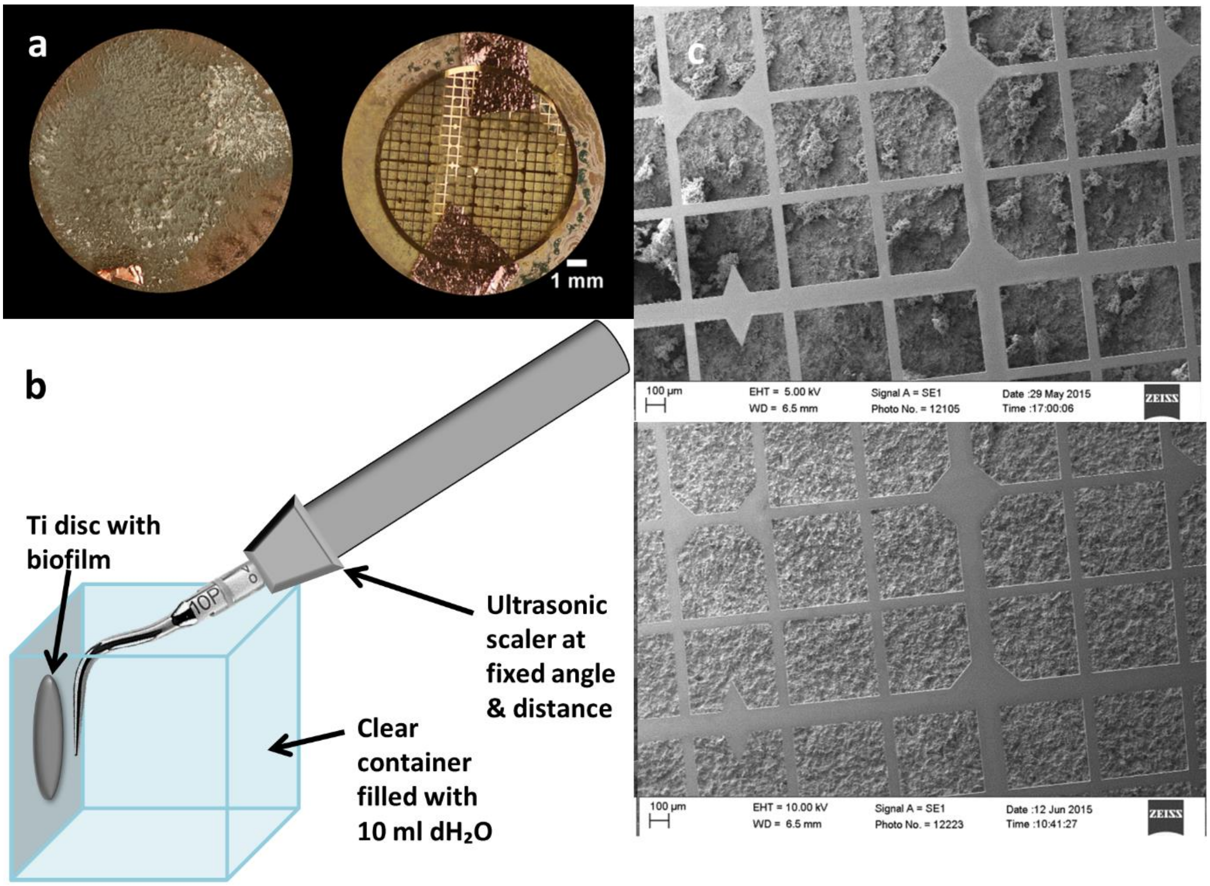

Supplementary Figure S1 (a) Photographs of the discs after biofilm growth and after applying the SEM finder grid for imaging. (b) Schematic showing experimental setup for biofilm removal experiments. (c) SEM images showing how the finder grids were used to locate the same area before and after removal.

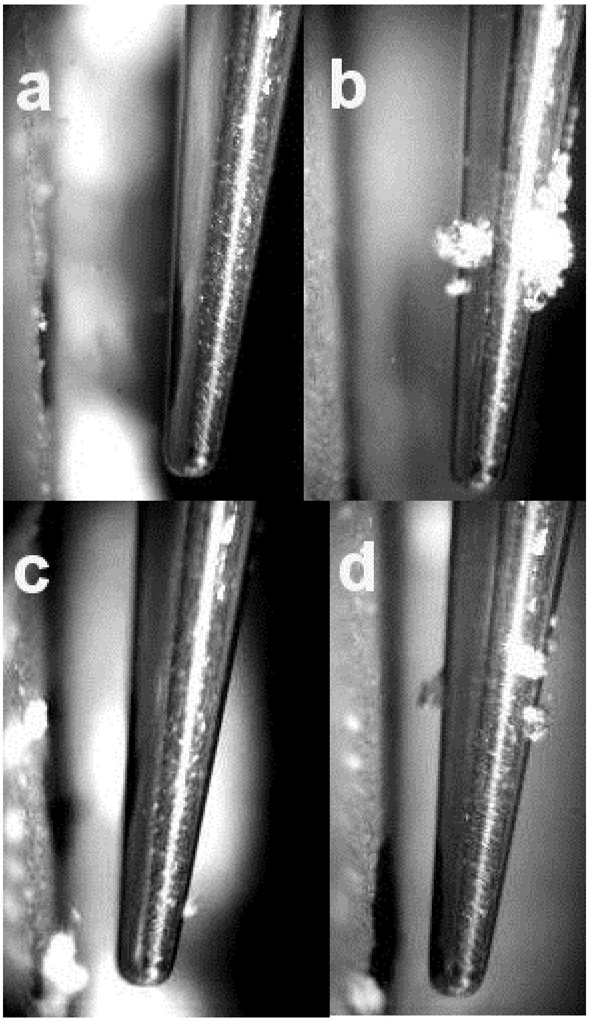

Supplementary Figure S2 High speed video stills of the ultrasonic scaler tip next to the sample during biofilm removal. (a) power 5, 1 mm away from disc. (b) power 10, 1 mm away from disc. (c) power 5, 0.5 mm away from disc. (d) power 10, 0.5 mm away from disc. Also see supplementary video S2. Videos taken at 128,000 fps and played back at 10 fps.
